# Triarylborane-Based Materials for OLED Applications

**DOI:** 10.3390/molecules22091522

**Published:** 2017-09-13

**Authors:** Gulsen Turkoglu, M. Emin Cinar, Turan Ozturk

**Affiliations:** 1 Department of Chemistry, Istanbul Technical University, Maslak, Istanbul 34469, Turkey; ecinar@itu.edu.tr; 2 Chemistry Group Laboratories, TUBITAK-UME P.O. Box 54, Gebze, Kocaeli 41471, Turkey

**Keywords:** triarylborane, fluorescence quantum yields, organic conjugated material, electroluminescence, OLED

## Abstract

Multidisciplinary research on organic fluorescent molecules has been attracting great interest owing to their potential applications in biomedical and material sciences. In recent years, electron deficient systems have been increasingly incorporated into fluorescent materials. Triarylboranes with the empty p orbital of their boron centres are electron deficient and can be used as strong electron acceptors in conjugated organic fluorescent materials. Moreover, their applications in optoelectronic devices, energy harvesting materials and anion sensing, due to their natural Lewis acidity and remarkable solid-state fluorescence properties, have also been investigated. Furthermore, fluorescent triarylborane-based materials have been commonly utilized as emitters and electron transporters in organic light emitting diode (OLED) applications. In this review, triarylborane-based small molecules and polymers will be surveyed, covering their structure-property relationships, intramolecular charge transfer properties and solid-state fluorescence quantum yields as functional emissive materials in OLEDs. Also, the importance of the boron atom in triarylborane compounds is emphasized to address the key issues of both fluorescent emitters and their host materials for the construction of high-performance OLEDs.

## 1. Introduction

**Figure 1:**
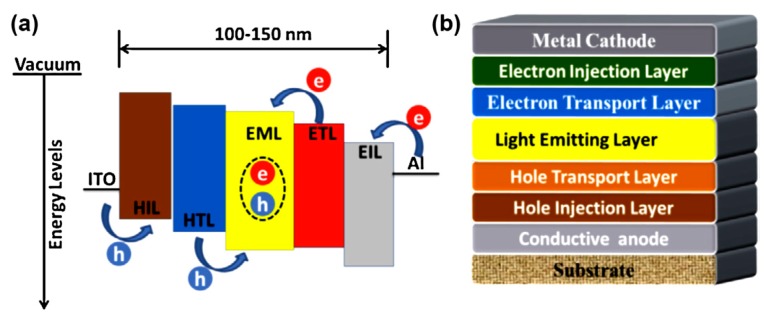
Typical device configuration of OLEDs. “Reprinted (adapted) with permission from Li, Y.; Liu, J.-Y.; Zhao, Y-D.; Cao, Y.-C. *Mater. Today* **2017**, *20*, 258–266. Copyright 2017 Elsevier [1]”.

Organic light emitting diodes (OLEDs), which are organic luminescent materials made of small molecules and polymers, have been widely used in next-generation high quality flat-panel displays and solid-lighting applications [1]. The operating principle of OLEDs is associated with electron-transporting (ETL) and hole-transporting layers (HTL), the interface of which functions as a site for the recombination and light emission processes depicted in Figure 1a together with typical device configuration in Figure 1b. High fluorescence quantum yields of luminous materials in the solid state, HOMO–LUMO energy levels, good electron and hole transport behaviours, film-forming properties, good thermal and oxidative stabilities, and outstanding colour purities are inevitable parameters for the construction of promising OLED devices [2]. In general, the external quantum efficiency (EQE) is the most direct parameter to define the device efficiency of OLEDs. Although, conventional OLEDs containing triarylborane-based materials as emitting layers have demonstrated low EQEs, their incorporation into OLEDs provided potential applications as efficient electron transporters [3,4] and electroluminescent (EL) layers [5,6,7]. Recently, EQEs, recorded for several devices containing new triarylboranes, have achieved the highest values up to 22.8% for green emission through the newly explored thermally-activated delayed fluorescence (TADF) process [8,9,10,11,12].

**Figure 2:**
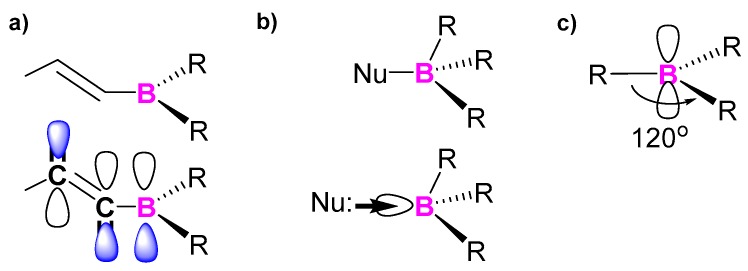
Three essential characteristic properties of boron atom for the molecular designs of new π-conjugated materials: (**a**) p_π_-π* conjugation; (**b**) Lewis acidity; and (**c**) trigonal planar geometry.

Triarylborane-based conjugated luminescent compounds have potential applications as important building blocks in material chemistry due to their excellent photophysical and electrochemical properties. They possess a trigonal planar sp^2^-hybridized trivalent boron atom with a vacant p orbital in the electronic ground state [13]. Thus, as triarylborane has an inherently electron deficient property, it is used as an electron acceptor and a Lewis acid in donor (D)–acceptor (A) organic π-conjugated systems. The steric hindrance around the boron centre, generated by the bulky groups, was proved several decades ago to be a useful way to prepare air-stable triarylboranes [13]. These compounds with broad π-conjugate systems render p_π_-π* conjugation effectively (Figure 2), which can be verified by their characteristic absorption and high emission properties [13]. Numerous interesting and highly functional triarylborane-based organic materials have been developed, including those with boron units as pendants [14,15,16,17,18] and in the main chain [19] of extended and conjugated materials. As a result of their photophysical properties, triarylborane-containing materials have been synthesized as promising units in diverse applications such as luminescent [20,21], electron-transporting [22,23], nonlinear optical and two-photon absorption materials [24,25] and anion sensors [26,27].

Furthermore, triarylboranes are highly luminescent materials in the solid state and they demonstrate electrochemically reversible one-electron reduction processes. In addition, their relatively lower unoccupied molecular orbital (LUMO) energy levels are a unique property [28,29,30]. Thus, the highest occupied molecular orbital (HOMO) levels of boron-based conjugated compounds are tunable with electron donor groups. When accompanied by electron donor units such as amines and carbazoles, the electron-accepting ability of boron centre is recognized to lead to low energy intramolecular charge transfer (ICT) transition bands upon photo-excitation, resulting in excellent solvatochromic fluorescence emissions [31]. Therefore, they have been investigated as highly efficient electron transporting and emissive materials in OLED devices [16,32,33,34,35]. Later, various research groups have reported a wide range of interesting electronic and optical properties of triarylborane possessing compounds and polymers [36]. Their applications in electronic and optical materials have expanded very rapidly over the last two decades [14,15,16,36,37]. In this review, we focus on triarylboranes as strong π-acceptors in D–A systems in terms of stability, structure-property relationship and high quantum efficiency in the solid state to realize potential applications in optoelectronic devices.

## 2. Triarylborane-Based Small Molecules/Oligomers

Small organoborane molecules have recently admitted increasing attention in optoelectronic devices as they have advantages such as easy synthesis and purification, flexible chemical modification and alternative film-forming process compared to polymer materials. Triarylboranes have been widely used as strong electron acceptors in D–A type materials for functional applications in optoelectronic devices. The presence of an inner strong electron donor group in such conjugated compounds produces extended LUMO over the whole molecule during the p_π_-π* conjugation and thus, crucially decreases its LUMO level, while much less effect was observed for HOMO [38,39,40]. Consequently, the decrease in LUMO energy influences the HOMO–LUMO gap and thus the absorption and emission properties of the compounds, providing boron-based π-conjugated systems with better electron acceptor quality [41]. Moreover, their high HOMO and low LUMO levels can enhance the carrier-injecting properties, improving the device performance. Therefore, smaller HOMO–LUMO gaps may lead to red/near infrared (NIR) emissive materials, which are rare for triarylborane-based compounds, particularly in the solid state [16,42,43,44]. Furthermore, triarylborane based D–A type molecules possess great electronic dipoles, which support hole–electron charge transfer depending on excitation [45,46].

**Figure 3:**
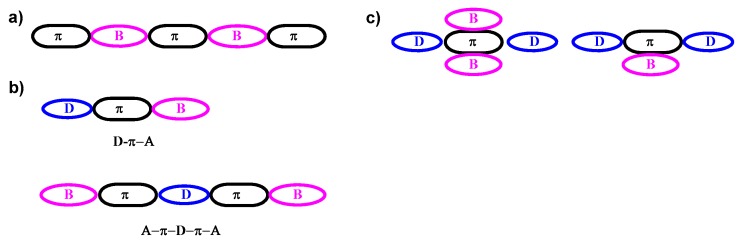
Schematic demonstration of π-conjugated D–A systems containing triarylborane groups (**a**) in the main chain; (**b**) at the terminal positions and (**c**) as a pendant in the side chain.

Several design strategies have been developed to understand how substituents in π-conjugated frameworks alter intrinsic properties, such as emission band, electron affinity, ionization potential, multiplicity of the emitting state and spatial arrangements of individual molecules in bulk [47]. Their presence in typical designs involve three structural basis: (1) essential fragment of π-conjugated systems in the main chain; (2) terminator located either at one terminal (D–π–A) or at both terminals (A–π–D–π–A) in π-conjugated systems; (3) pendant connected to the π-conjugated framework (Figure 3) [15,37,47,48].

### 2.1. Triarylborane-Based Conjugated Systems Having Boron in the Main Chain or as A Terminator

**Figure 4:**
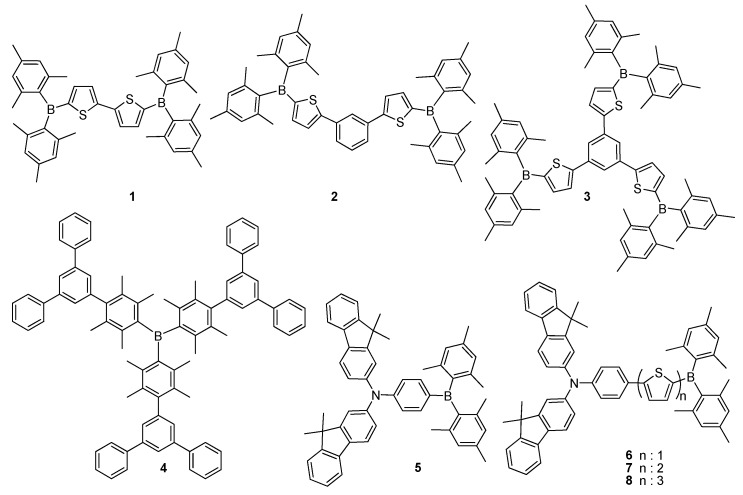
Chemical structures of conjugated D–A type materials **1**–**8**.

The empty p-orbital of boron can enable efficient electronic delocalization with linked π-conjugated units. Therefore, borane-functionalization approaches usually supply molecules with important ICT character due to the effective electronic coupling between the empty p-orbital of boron and the π-orbitals of electron donor units. In general, these materials are either symmetric on both sides of the boryl group (A–D–A type) or they are combined with an electron-donating group (D–π–A type), thus, they display strong fluorescence properties with large Stokes shifts. However, most of the linear organoboranes utilize dimesitylboron (Mes_2_B), in which two mesityl substituents are introduced on the trivalent boron centre mainly to provide enough steric protection. The use of A–D–A type conjugated material **1**, possessing triarylborane and bithiophene units, was investigated by Shirota and co-workers, who indicated their potential applications as blue emitters and electron-transport materials in OLEDs (Figure 4) [49]. They also reported linear **2** and star-shaped **3** molecules, respectively, containing bi- and tri substituted benzene and, thiophene as π-spacers and dimesitylboron (Mes_2_B) as an electron acceptor unit (Figure 4) [4]. These compounds were examined owing to their functional applications as efficient electron transporters emerging from multiple redox properties in electrochemical reduction as well as their thermally stable amorphous behaviours. Especially, the device prepared using compound **3** demonstrated an effective electron-transporting hole blocker property for blue-emitting organic EL devices with a maximum luminance (*L_max_*) of 4130 cd·m^−2^ at a driving voltage of 17 V, a luminous efficiency of 0.48 lm·W^−1^ and an EQE of 0.8% at a luminance of 300 cd·m^−2^. Later, the same group investigated a series of triarylborane-based materials containing duryl groups to function as hole blockers in organic EL devices [50]. These compounds demonstrated reversible reduction peaks with high thermal stabilities and relatively high HOMO and LUMO energy levels. Among these compounds, **4** as a hole blocker, exhibited the highest performance with a *L_max_* of 9100 cd·m^−2^ at a driving voltage of 11 V and EQE of 2.5% in blue emitting OLED devices (Figure 4).

In order to improve the performance of OLED, Shirota’s group synthesized triarylborane-based ambipolar D–A type molecules **5**–**8** as new colour-tuned emitting materials for EL applications (Figure 4) [6]. Due to their ambipolar characters, they displayed reversible oxidations and reductions in cyclic voltammetry. They emitted greenish-blue, green, yellowish-green, and light yellow in organic EL devices, colours of which were tuned by varying the conjugation length of the central thiophene units. The conventional OLED device, fabricated using **5** as a host material for emissive dopant, exhibited the highest performance with a *L_max_* of 35,740 cd·m^−2^ and luminous efficiency and EQE of 4.3 lm·W^−1^ and 2.1% at a luminance of 300 cd·m^−2^, respectively. Moreover, Yu and co-workers used **5** as both hole-electron transporting and green light-emitting layer for white organic light-emitting diode (WOLED) device [51]. A quite pure WOLED with Commission Internationale De L’Eclairage (CIE) values of 0.33 and 0.36, comprising three main colours (blue, green and red) was attained with the optimized device. While a ultrathin red fluorescent material of 3-(dicyanomethylene)-5,5-dimethyl-1-(4-dimethylamino-styryl)cyclohexene (DCDDC) layer was inserted in **5**, a 4,4′-bis(2,20-diphenyvinyl)-1,10-dipenyl (DPVBi) layer was utilized as a blue light emitting layer [50,51]. Also, an OLED device, based on compounds **4** and **7**, emitted white light displaying high performance with a *L_max_* of 16,600 cd·m^−2^ at 11.0 V, luminous efficiency and EQE of 1.2 lm·W^−1^ and 0.90% at a luminance of 300 cd·m^−2^, respectively [47,48,49].

**Figure 5:**
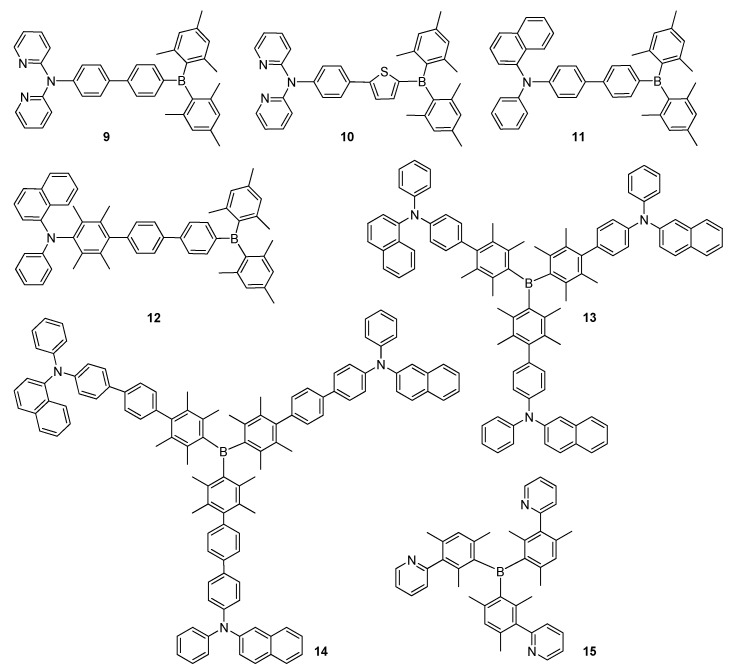
Triarylborane-based conjugated D–A type materials **9**–**15**.

Wang and co-workers reported a series of new D–A type small molecules containing Mes_2_B as an electron acceptor with dipyridylamine and 7-azaindolyl-functional aryl/thienyl as an electron donor unit for OLED applications [52]. Among these compounds, molecules **9** and **10** were used as blue emitters, displaying high fluorescence quantum yields (*Φ_F_*) of 63% and 100% in dichloromethane and strong emission bands at 440 and 469 nm in solid state, respectively (Figure 5). Their optimized OLED devices exhibited moderate performances with maximum brightness of 2566 cd·m^−2^ at 17 V and 1510 cd·m^−2^ at 24 V, respectively. Wang and co-workers synthesized a novel luminescent ambipolar molecule (**11**) containing (1-naphthyl)phenylamino group, which was widely employed as a hole transporting material in OLEDs (Figure 5) [53]. Molecule **11** provided a bright blue emission with a maximum at 452 nm and a luminescent quantum yield of 31% (*Φ_F_* = 95% in THF) in solid state and a strong solvatochromic emission behaviour. A triple-layer device fabricated using molecule **11** as an emitting material, which displayed a maximum brightness of 5053 cd·m^−2^ at 12 V with a maximum efficiency of 6.0 cd·A^−1^ at 8 V. Later, for an optimized device based on molecule **11** as an undoped blue emitter, a maximum current, power efficiency and brightness were achieved as high as 3.5 cd·A^−1^, 2.5 lm·W^−1^ and 7000 cd·m^−2^ at 10 V, respectively, and 200 cd·m^−2^ at 4.5 V [32]. Molcules **12**–**14** containing duryl and (1-naphthyl)phenylamino as electron donor units displayed termal stability, high emission quantum efficiency, strongly solvatochromic effect, characteristic D–A intramolecular charge transfer and a highly polarized excited state (Figure 5) [54]. Among the optimized devices, the device constructed using compound **14**, which could be used as a hole transporter and a hole injection material, was found to be the most promising one.

The pyridine substituents in compound **15** endow this material with a high triplet energy electron transport property required in blue and deep blue phosphorescent OLED applications (Figure 5) [55]. For compound **15**, a high quantum efficiency was recorded owing to suppressed triplet exciton quenching compared to the other electron transport materials in blue phosphorescent OLEDs [56]. The device performances of deep blue phosphorescent OLEDs based on **15** exhibited a maximum current efficiency (η_C,max_) of 60 cd·A^−1^ at a luminance of 500 cd·m^−2^ and high electron mobility of 10^−5^ cm^2^·V^−1^·s^−1^ [56].

**Figure 6:**
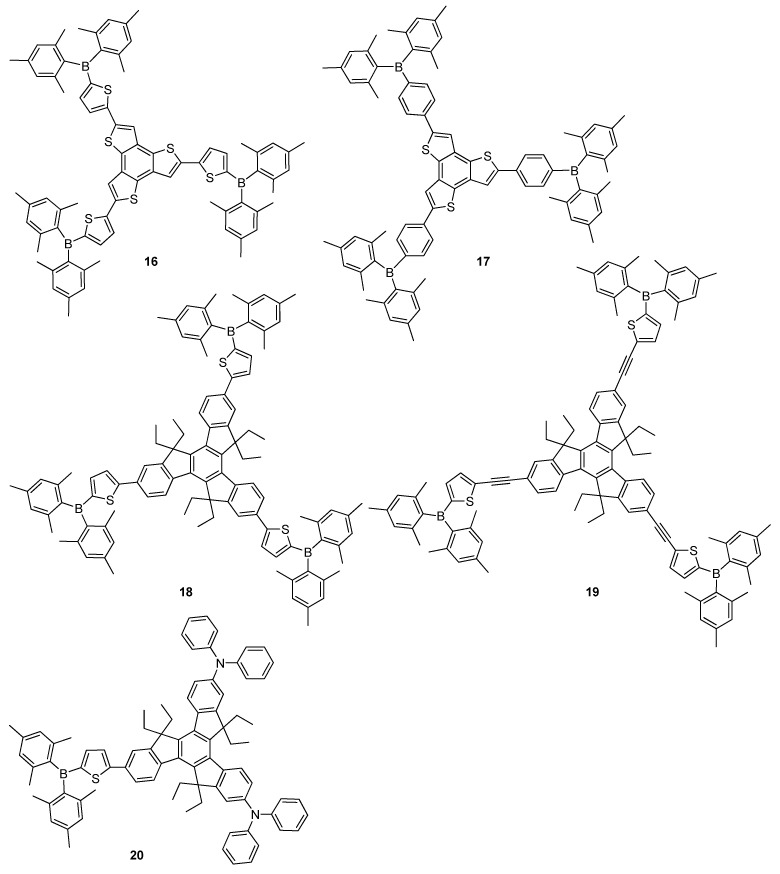
Triarylborane-based octupolar π-conjugated materials **16**–**20**.

Furthermore, linear and star-shaped triarylborane compounds also demonstrated ICT properties. Multi-branched π-conjugated frameworks having large building blocks were used to obtain high emissive organic materials in both solution and solid state. Liu and co-workers investigated fluorescent dipolar [57], quadrupolar [58] and octupolar multi-branched triarylborane moieties as optoelectronic functional materials [59]. Octopolar multi-branched **16** and **17** had terthienobenzene as an electron donor and Mes_2_B as electron an acceptor (Figure 6) [59]. These compounds demonstrated strong blue-green emission in common organic solvents as well as good thermal stabilities and relatively high *Φ_F_* of 33% and 40%, respectively, which were considered to be promising materials in optoelectronic applications. Recently, Wang and co-workers reported triarylborane-terminalized branched π-conjugated compounds **18**–**20** containing a truxene core as a large building block (Figure 6) [60]. They exhibited blue emissions appearing at 440, 447 and 499 nm with high luminescence quantum efficiencies of 67%, 60% and 43% as thin films, respectively. Also, compounds **19** and **20** showed excellent *Φ_F_* of 79% and 57% in powder form, representing their potential applications as outstanding non-doped luminescent materials.

**Figure 7:**
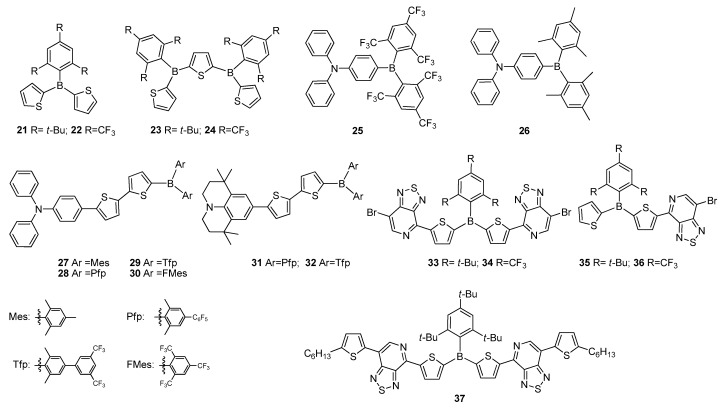
Triarylborane-based materials **21**–**37**.

Triarylborane plays two roles in organic electronic applications, namely: (i) stability enhancement and (ii) providing the material with strong electron acceptor character. The widely used bulky mesityl groups are generally provided to protect them against air, water and most nucleophiles [16,61,62]. Furthermore, several other groups have also been reported to be applied to stabilize tricoordinate boranes, such as 2,4,6-tri-isopropylphenyl (Tip) [63], 2,4,6-tri-*tert*-butylphenyl (Mes*) [64] and 2,4,6-tris(trifluoromethyl)phenyl [65]. The use of different bulky units were reported by Jäkle and co-workers, who synthesized a series of oligomers **21**–**24** containing thiophene as electron donor unit with aryl boron as an electron acceptor moiety in the presence of highly bulky 2,4,6-tri-*tert*-butylphenyl (Mes*) and 2,4,6-tris(trifluoromethyl)phenyl (FMes) groups (Figure 7) [65]. These compounds demonstrated resistance against air and moisture, and even against acid and strong bases. Moreover, the strong electron-withdrawing FMes groups of **22** and **24** increase electron acceptor properties of boron atom. Thus, both HOMO and LUMO energy levels of **22** were significantly lowered. Among them, **24** showed only a very weak blue fluorescence with a quantum yield of 0.8%, while displaying a slightly better fluorescence with a *Φ_F_* of 1.8% in thin film.

The “push–pull” structures possessing an electron-accepting triarylboron group and an electron-donating amino unit resulted in ICT transitions, which usually provided interesting fluorescence properties with solvatochromic effects. Marder and co-workers introduced a series of compounds containing strong electron-acceptor FMes group into D–π–A systems [66], among which **25** was observed to have the strongest acceptor character, leading to significantly red-shifted emissions in solution and the solid state, stronger emission solvatochromism and notably lower reduction potentials compared to the Mes-substituted derivative **26** (Figure 7). The same group separately reported D–π–A type materials containing triphenylamine (TPA) and 1,1,7,7-tetra-methylijuloidine as electron donor units, bithiophene as a π-bridge spacer and triarylboron substituted mesityl ((Mes)_2_B), 2,6-dimethyl-4-pentafluoropenylbenzene((Pfp)_2_B), 2,6-dimethyl-4-[3,5-bis(trifluoromethyl) phenyl]benzene ((Tfp)_2_B) and 2,4,6-tris(trifluoromethyl)benzene ((FMes)_2_B) as electron acceptors [67]. Comprehensive photophysical and electrochemical investigations of **27**–**32** indicated that electron-accepting ability increased in the order of (Mes)_2_B < (Pfp)_2_B ≈ (Tfp)_2_B << (FMes)_2_B (Figure 7). Compounds **27**–**29** showed yellow emissions at 548, 545 and 563 nm in powder form, while **30**–**32** displayed red emission at 646, 635 and 618 nm, and these compounds exhibited *Φ_F_* ranging between 0.46 and 0.05 in solid state, respectively, which were much lower than those recorded in their solutions. They exhibited efficient green to red (*Φ_F_* = 0.80–1.0) emissions depending on the solvent polarity. Also, intense near infrared (NIR) emission was achieved with derivatives **31** and **32**, containing moderately strong acceptors (Pfp)_2_B and (Tfp)_2_B, and a strong donor 1,1,7,7-tetramethylijuloidine units (*Φ_F_* = 0.27–0.48). The above characteristics allowed them to be considered as red emitters and electron transporting materials.

Jäkle and co-workers introduced a series of triarylborane compounds **33**–**36** functionalized with a second electron acceptor, pyridyl[2,1,3]thiadiazole (Figure 7) [68]. These compounds exhibited high stability and were highly photoluminescence (PL) having emission bands in the range of 495 to 535 nm with *Φ_F_* values between 32% and 52% in solution. They indicated coherent red shift with emission bands appearing between 578 and 622 nm, and compounds **33** and **34** rendered relatively high luminescence quantum yields of 12% and 18% in solid state, respectively. Extension of conjugation in **37**, synthesized via coupling of **33** with 2-hexylthiophene, resulted in higher thermal stability and solubility [68]. Compound **37** demonstrated strong red-emission at 610 nm with high *Φ_F_* of 68%, and an excellent electrochemical reversibility compared to **33**.

**Figure 8:**
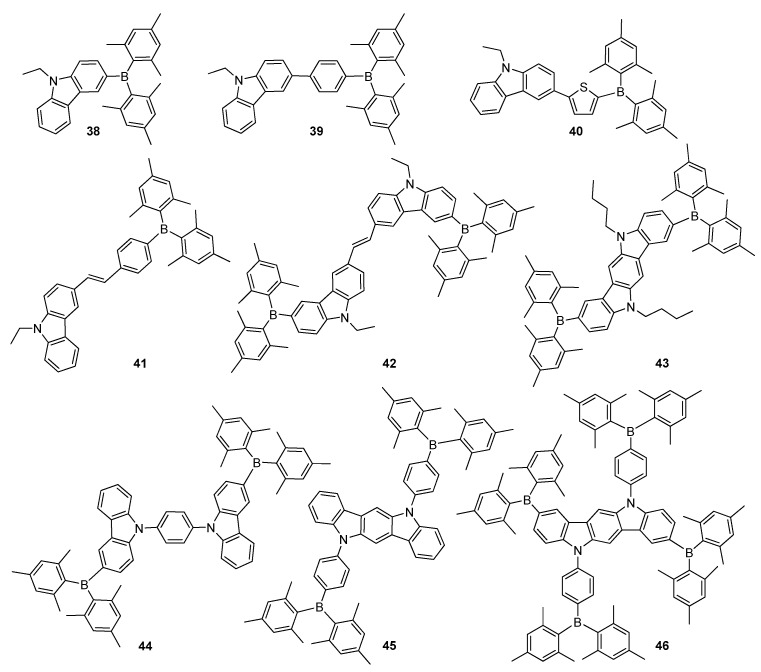
Carbazole possessing triarylborane-based materials **38**–**46**.

Moreover, triarylborane-based compounds have been widely utilized as blue emitting materials, which are essential ingredient for full colour displays due to their high brilliance and obvious colour purities [42]. Although blue OLEDs required the use of a dopant system to improve OLED efficiency, control of the dopant concentration using co-evaporation methods was not an easy task. Thus, this fabrication problem was overcome by the development of high performance blue light-emitting materials, which are suitable for non-doped blue OLEDs. Ambipolar blue light emitting materials with the components of electron-donors (carbazole, etc.) and electron-acceptor such as triarylborane ease exciton formation via stable cation and anion radicals, and improve charge balancing together with EL efficiency in OLEDs [5,6,69]. Carbazoles are known to produce short wavelength absorption and emission spectra due to their weak electron donor character with respect to other amino-donors [70]. Therefore, Jeng and co-workers synthesized ambipolar fluorophores **38**–**41** containing carbazole as electron donor, different π-spacer groups and Mes_2_B for potential applications in highly efficient non-doped blue OLEDs (Figure 8) [71]. Compounds **38**–**41** displayed deep-blue to greenish-blue PL spectra with emission peaks at 403, 443, 470 and 503 nm, respectively, among which **39** and **40** had lower LUMO energy levels and blue photoluminescent solid state quantum yields as high as 54% and 51%, respectively. While the OLED based on **39** was a deep-blue with the CIE coordinates of (0.15, 0.09), EQE of 4.3% and maximum brightness of 5350 cd·m^−2^, the device fabricated using **40** demonstrated blue EL with the CIE coordinates of (0.13, 0.21), higher EQE of 6.9% and maximum brightness of 28,300 cd·m^−2^. This state can be rationalized by their relatively high solid state *Φ_F_* of **39** and **40**, which are also crucial to achieve high EQEs. Moreover, the ideal arrangement of HOMO–LUMO energy levels in devices also contributes significantly.

A new A–D–π–D–A type fluorescent material **42**, bearing carbazole units as electron donors and Mes_2_B units as electron acceptors was synthesized by Shi and co-workers (Figure 8) [72], and demonstrated a high oxidative stability, strong fluorescence solvatochromism properties and a high *Φ_F_* of 0.81 (in *n*-hexane), thereby, exhibiting promising potential applications in OLEDs. The same group reported a new linear material **43**, prepared by linking triarylborane to both terminals of indolo[3,2-*b*]carbazole (ICZ). Its large planar and rigid conjugated structure was expected to improve the efficiency and stability in OLED application (Figure 8) [73]. It displayed a blue emission band at 452 nm and a high *Φ_F_* of 0.76 in solution as well as excellent thermal and electrochemical stabilities. The OLED device constructed using **43** as an emitting material exhibited pure blue emissions at different voltages and relatively good EL performances with a turn-on voltage (V_on_) of 3.6 V and a maximum luminance efficiency (η_L,max_) of 1.67 cd·A^−1^ and a *L*_max_ of 5342 cd·m^−2^. The same group separately synthesized a new fluorescent ambipolar material **44**, containing Mes_2_B and phenylcarbazole moieties (Figure 8) [74], which exhibited blue emission of luminescence maximum wavelength at 480 nm with excellent thermal and electrochemical stabilities as well as aggregation induced emission (AIE) property [75]. Moreover a multi-layer device based on **44** demonstrated good EL performances with V_on_ of 3.8 V, η_L,max_ of 3.25 cd·A^−1^ and *L*_max_ of 2784 cd·m^−2^ with blue CIE coordinates (0.23, 0.35) in non-doped OLED. Shi et al. reported branched and star-shaped materials **45** and **46**, containing two and four Mes_2_B substituents and planar ICZ core unit, respectively (Figure 8) [76]. These compounds showed high *Φ_F_*, strong ICT, good electrochemical and electron transporting properties. The OLED device based on **45** exhibited blue emission (λ_EL_ = 472 nm) with a V_on_ of 6.1 V, *L_max_* of 5634 cd·m^−2^ and η_L,max_ of 2.96 cd·A^−1^, whereas the device using **46** demonstrated electroluminescence emission peak appearing at 8 nm shorter compared to **45** (464 nm) with V_on_ of 3.6 V, *L_max_* 2036 cd·m^−2^ and η_L,max_ of 2.88 cd·A^−1^.

**Figure 9:**
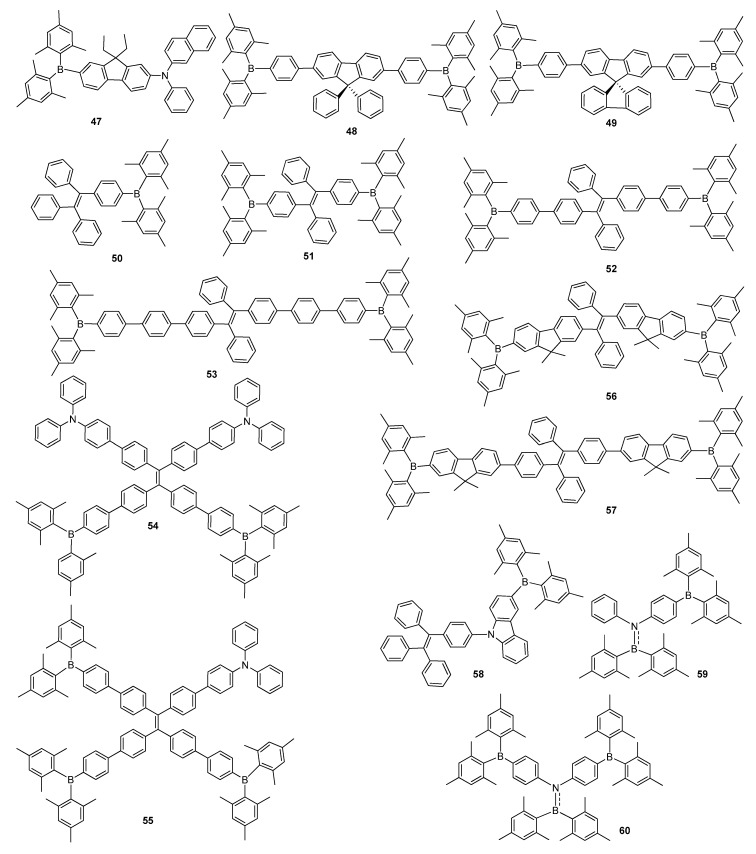
Triarylborane-based florescent materials **47**–**60**.

Fluorene has also been employed to construct boron-based emitting materials. A fluorene-bridged compound with a fused ring system greatly enhanced the thermal stability and charge-transporting capability of the material [77]. Zhao and co-workers realized asymmetrical ambipolar material **47** by linking α-naphthylamino and triarylborane groups via a fluorene bridge (Figure 9) [77], five fabricated devices of which exhibited blue to green emissions between 456 and 528 nm as multifunctional compound in OLED applications. A four layer device of **47** indicated a better performance with *η*_C,max_ of 5.03 cd·A^−1^ and blue emission with CIE of (0.19, 0.31) compared to other device applications. The properties of the devices indicated that **47** as a multifunctional optoelectronic material can be utilized as a blue emitter and hole- and electron-transporting material. Xu et al. reported **48** and **49**, possessing fluorene derivatives and Mes_2_B as deep blue emitting materials (Figure 9) [78]. They showed deep blue emission peak at ~430 nm with excellent *Φ_F_* of up to 94% as thin film and low-lying LUMO energy levels, which indicated their excellent properties allowing them to be used as both emitter and electron transporting materials. The optimized device based on **48** achieved efficient deep blue performance with EL maximum at 432 nm (CIE = 0.179, 0.128), *L_max_* of 10,320 cd·m^−2^ and η_C,max_ of 3.4 cd·A^−1^ compared to the *η*_C,max_ of 2.7% recorded from the device with **49**. These results indicated them as deep blue fluorescent emitters with good electron-transporting abilities arisen from the presence of a strong Mes_2_B acceptor.

Tetraphenylethene (TPE), used to enhance the emission of materials upon formation of aggregates, functioning according to AIE [75], was utilized as a part of functional materials in OLED applications due to improved solid state emission efficiency and a high hole transporting ability. Yuan et al. synthesized highly fluorescent material **50** containing TPE unit and Mes_2_B group, displaying notable EL performance, high thermal stability, AIE activity with a solid state emission efficiency of 100% and good electron-transport property (Figure 9) [21]. A non-doped EL device constructed using **50** exhibited new bluish-green emission with *η*_C,max_ of 7.13 cd·A^−1^ and EQE of 2.7%. Chen et al. reported A–π–D–π–A type luminescent symmetrical materials **51**–**53**, possessing TPE as π-conjugated bridge substituted by two Mes_2_B terminal groups (Figure 9) [79]. All the materials had AIE features and highly fluorescent quantum yields of 81–86% as solid films. According to the electron deficient nature of the boron atom and very low LUMO energy levels of **51**–**53**, they were used as both good electron transporting and high light emitting layers in OLED devices. The A non-doped OLED device based on **51** exhibited the best device performance with *η_C,max_* of 13.5 cd·A^−1^ and EQE of 4.6%, while **52** and **53** based devices showed moderate EL efficiency of 5.1 cd·A^−1^ and EQE of 2% and 2.6%, respectively.

Tang and co-workers comprehensively investigated a series of novel star-shaped bipolar emitters **54** and **55**, containing TPE as an AIE active unit with different proportions of TPA as a donor and Mes_2_B as an acceptor groups (Figure 9) [80]. Their dim *Φ_F_* of 2.8–4.5% in solution increased up to 91–95% in solid state, indicating AIE characteristics. Owing to their good solution-process film-forming abilities, devices containing AIE materials were constructed for non-doped OLED applications. A device fabricated with **54** demonstrated the best efficiency with *L_max_* of 11,665 cd·m^−2^, *η_C,max_* of 8.3 cd·A^−1^, EQE of 2.6% with CIE at 0.37, 0.54, whereas compound **55** based EL device gave green emission of CIE at 0.35, 0.53 with η_C,max_ of 6.3 cd·A^−1^. The same group reported AIE-active materials **56** and **57** bearing TPE with fluorenyl group and Mes_2_B moiety (Figure 9) [81]. The combination of a fluorenyl group with a Mes_2_B unit displayed significantly augmented molecular thermal stabilities and excellent solid state emission efficiencies, as a result of lower LUMO energy levels. The multilayer non-doped OLED device based on **57** as a light-emitting layer achieved high EL performance as a sky-blue emitter with η_C,max_ of 12.2 cd·A^−1^ and EQE of 5.3%, ultrahigh brightness of 92,810 cd·m^−2^ and low efficiency roll-off of 11.0 cd·A^−1^ at 1000 cd·m^−2^. These results pointed out that TPE-functionalized triarylborane materials could be considered to be crucial important for the commercial applications of OLEDs.

Choi’s group reported luminescent material **58** bearing a TPE unit connected to a carbazole core as a hole transporting unit and Mes_2_B as electron transporting group to develop the blue light-emitting material (Figure 9) [82]. Compound **58** was indicated to be a promising emitting material for non-doped EL devices, owing to its excellent thermal, electrochemical and charge-transporting properties. Its OLED device furnished pure blue emission appearing at 464 nm with CIE coordinates of (0.18, 0.21), *L*_max_ of 4624 cd·m^−2^ and *η_C,max_* of 4.28 cd·A^−1^. Thilagar’s groups reported new polyaromatic aminoboranes **59** and **60** containing substituted one or two Mes_2_B units (Figure 9) [83]. These compounds exhibited pure blue emissions with AIE enhancement (AIEE) features in solid state, rendering to be used as promising materials for potential applications in blue OLEDs.

**Figure 10:**
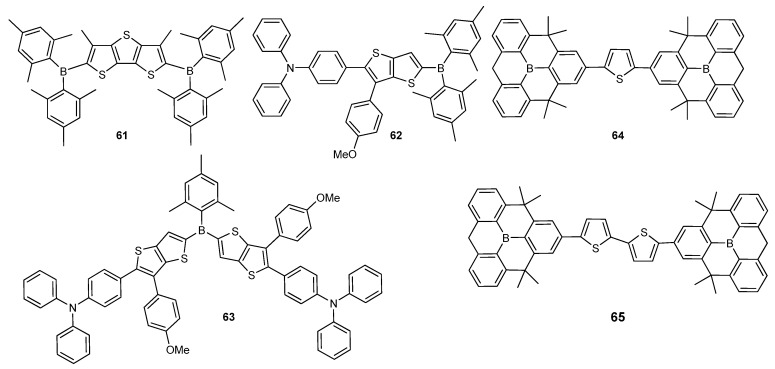
Triarylborane-based materials **61**–**65**.

Fused thiophene units are one of the most popular building blocks utilized in organic π-conjugated materials [84,85,86]. The first potential application in OLEDs was achieved using dithienothiophene (DTT) as an electron donor with triarylborane terminal groups (**61**) in 2005 by Mazzeo et al. (Figure 10) [87]. The superposition of the intrinsic blue-green light emission (λ_em_ = 480 nm) with an additional narrow red-shifted emission at 680 nm in solid state provided white EL property. Due to the potential effect on the lighting industry and backlight applications, white-light emission generated from organic compounds has been the subject of growing interest. A low-energy peak at 680 nm in EL spectrum was more intense than in PL spectrum, showing that a more effective population in aggregation state happened upon electrical injection. A bright single-layer white OLEDs by spin-coating a single emitting material **61** exhibited a clear white-light emission with CIE coordinates of 0.31, 0.42 with a *L*_max_ of 3800 cd·m^−2^ and EQE of 0.35%, opening new avenue for the construction of low-cost single-active material white OLEDs. Replacement of DTT with 2,5-dithienyl substituted thieno[3,2-*b*]thiophene (TT) and thieno[2,3-*b*]thiophene in **61** was realized by Turkoglu et al. who reported *Φ_F_* of 38% and 53% in THF as green and blue emitters, respectively [86]. Recently, Ozturk and co-workers comprehensively investigated D–A and D–A–D type small materials **62** and **63** bearing electron donor TPA linked through a methoxyphenyl substituted TT π-conjugated spacer with triarylborane for OLED applications (Figure 10) [88]. Both compounds showed high *Φ_F_* of 46% and 68% in THF, and their PL quantum yields were recorded up to 42% in solid state. Furthermore, they demonstrated strong positive solvatochromisim consistent with an ICT transition over a B/N D–π–A system. The OLED device based on **62** as an emissive material exhibited a bright green emission (λ_EL_ = 500 nm) with CIE coordinates of (0.16, 0.51) and a luminous efficiency of 0.40 cd·A^−1^ with EQE of 0.15%. Also the device constructed using **63** doped with **62** showed a yellowish-green emission at 534 nm, and exhibited more efficient device performance with η_L,max_ of 0.45 cd·A^−1^, compared to its non-doped device.

Moreover, boron is also used as a dopant as it forms electron poor and easily reducible π-electron systems. In addition, polycyclic aromatic compounds with boron, generally demonstrating bright luminescence, are the most important scaffolds for organic optoelectronic materials [89,90,91]. To indicate the potential of planar boranes to be utilized as building blocks as electron-transporting materials in OLEDs, Yamaguchi and co-workers reported π-extended planar triphenylboranes connected via thiophene and bithiophene as a π-conjugated spacer in **64** and **65** (Figure 10) [89]. Compound **64** exhibited a blue emission appearing at 441 nm with high *Φ_F_* of 92% in solution and showed bathochromic shift with λ_em_ of 471 nm in solid state as well as excellent electrochemical properties, whereas **65** gave greenish fluorescence emission with λ_em_ of 482 nm (*Φ_F_* = 25%) and a green luminescence peak at 531 nm in solid state due to extension of π-conjugation on account of bithiophene unit. The OLED devices having these compounds as electron-transporting materials were successfully fabricated by a vacuum vapour deposition technique.

**Figure 11:**
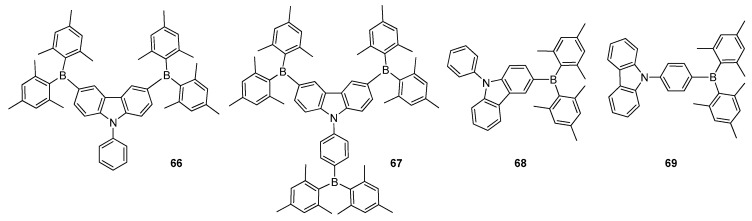
Triarylborane based phosphorescent materials **66**–**69**.

Organic EL materials are regularly categorized into fluorescent and phosphorescent materials depending on their luminescence mechanisms. Phosphorescent materials have become popular as OLED materials alternative to fluorescent compounds due to their excellent light-emitting performances [92]. Recently, ambipolar molecules containing electron donors such as carbazole, arylamine and electron acceptors such as triarylboranes have appeared as attractive materials for organic EL phosphorescent devices as a result of their excellent luminescent, hole-transporting and electron transporting properties. Lin et al. reported a new ambipolar host material **66** having two Mes_2_B and carbazole core units for phosphorescent OLED applications (Figure 11) [93]. This compound exhibited high EQEs of 20.7% for red, 20.0% for green, 16.5% for blue, and 15.7% for white emissions. Later, Shi and co-workers connected a Mes_2_B group symmetrically to a phenylcarbazole at the *para* position in molecule **66** to obtain new star-shaped host material **67**, which demonstrated excellent thermal and electrochemical stabilities, high *Φ_F_* of 0.95 (in THF) and high triplet energy (2.83 eV) (Figure 11) [94]. Various colours of phosphorescent OLED devices constructed using **67** exhibited a maximum brightness of *η_C,max_* of 12,337 cd·m^−2^ and 11.04 cd·A^−1^ for red, 26,473 cd·m^−2^ and 38.60 cd·A^−1^ for green and 7622 cd·m^−2^ and 7.39 cd·A^−1^ for blue at different voltages. As a result, it demonstrated great potential in generating phosphorescent OLED devices for display and lighting applications.

Recently, Jang et al. developed two structural comparable isomers of diarylboron-substituted phenylcarbazole as ambipolar host materials **68** and **69** to examine their green phosphorescent OLED performances (Figure 11) [95]. Surprisingly, their HOMO and LUMO levels were found to be appropriate for hole and electron injections, which supplied high hole and electron current densities with better charge transport properties. Moreover, the triplet energies of **68** and **69** were calculated as 2.88 eV and 2.72 eV, indicating their potential use as host materials in green phosphorescent OLEDs. While the device based on **69** exhibited better performance with EQE of 23.8% with a green colour coordinate of (0.30, 0.63), the device with **68** did not render a better excellent outcome but only EQE of 6.5% at 1000 cd·m^−2^.

**Figure 12:**
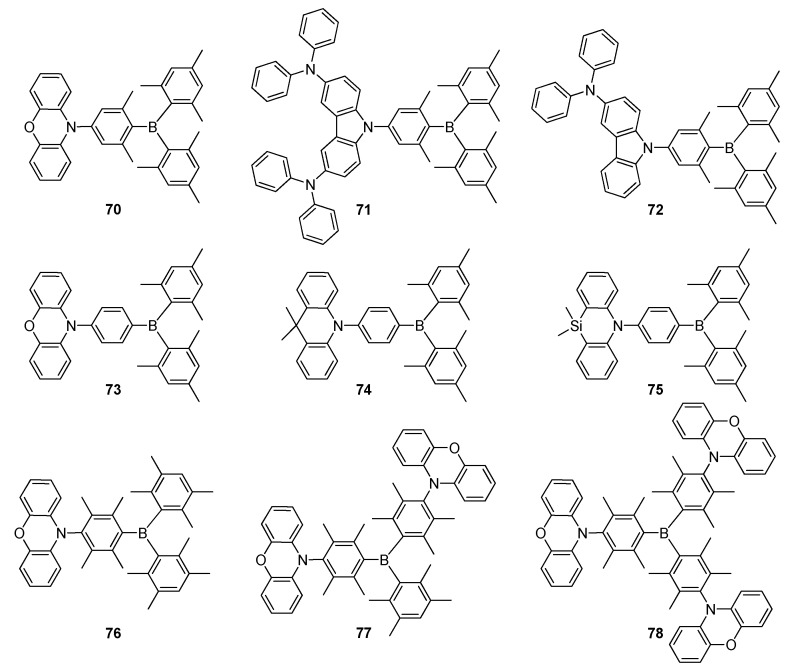
Triarylborane-based TADF materials **70**–**78**.

In recent years, TADF materials have also received much attention as highly efficient emitters for OLEDs with high EQEs [8,96]. TADF emitters, generally containing D–A type systems, work based on reverse intersystem crossing (RISC) involving conversion of the lowest triplet excited state (T_1_) to the lowest singlet excited state (S_1_) by thermal activation [8,94,97]. The most important requirement for TADF emission is a very small singlet-triplet energy gap (Δ*E*_ST_) which promotes RISC [10,95,98]. There are only few reports about the investigation on triarylborane-based TADF materials due to the newly expanding area of TADF [8,9,99]. Some of them are briefly discussed here. Kaji and co-workers reported triarylborane-based TADF emitters **70**–**72**, having electron-acceptor trimesityl-boron derivatives and various amine-based electron donor units (Figure 12) [10]. All the compounds had low Δ*E*_ST_ values of 8.0, 21 and 19 meV, respectively, achieving high PL quantum yields. Doped OLED using **71** as an emitter showed a sky-blue emission with EQE of 21.6%, which is one of the highest values for a TADF-based blue OLEDs. Surprisingly, the OLED device with **70** as an emitter dopant exhibited green emission with CIE coordinates of 0.22, 0.55 and EQE of 22.8%, which is the highest value for an OLED with a triarylborane-based emitter to date. Kitamoto et al. reported new D–A materials **73** and **74** containing 9,9-dimethylacridane and phenoxazine as electron-donating units linked to dimesitylphenylborane as an electron-acceptor, which OLED devices exhibited TADF characteristics (Figure 12) [100]. Their Δ*E*_ST_ values were estimated to be 41 and 33 meV, and their PL quantum yields were recorded to be 89% and 87% in toluene, respectively. The OLED devices using **73** and **74** exhibited light blue with EQE of 16.0% for **73** and green emission with EQE of 17.3% for **74**. The same group replaced sp^3^ carbon with silicon atom to obtain **75**, which showed pure blue emission with a peak wavelength of 462 nm and CIE coordinates of (0.14, 0.15) (Figure 12) [101]. **75** showed pure blue emission with a peak wavelength of 462 nm and CIE coordinates of (0.14, 0.15). It demonstrated a relatively low *ΔE*_ST_ value of 57 meV with high PL quantum yield of 81%.

Yang and co-workers prepared a series of new triarylborane-based compounds **76**–**78** composed of a phenoxazine electron donor with a boron acceptor, which were linked via a highly sterically hindered duryl groups (Figure 12) [102]. Interestingly, all the compounds presented small Δ*E*_ST_ (120 meV, 50 meV, and 10 meV) and high PL quantum yields up to 95%. The best solution processed OLED device using **78** reached efficient green emission with *η_C,max_* of 41.5 cd·A^−1^, power efficiency of 32.6 lm·W^−1^ and EQE of 13.9%, which are among the highest for the solution processed OLEDs, assembled using boron-containing emitters. Furthermore, the devices based on **76** and **77** demonstrated bluish-green emissions at 480 and 496 nm with EQEs of 1.0% and 8.9%, respectively.

### 2.2. Triarylborane-Based Conjugated Systems Possessing Boron Pendant in the Side Chain

Small and oligomer molecules containing triarylborane moieties as pendants were expected to be an alternative approach that would exhibit different chemical and electronic properties [15]. These structures present an alternative pathway for electronic coupling between the empty p-orbital of the boron centre in the side chain and the π-orbitals of the main-chain. The achievement of this molecular design was recognized with two different effects of side chain arylboryl groups, namely, the steric bulkiness and an electron-accepting ability [16,44,46,103,104]. While the first one suppresses the intermolecular interactions, the second one brings about a large Stokes shift as a result of ICT transitions, and thus, results in efficient solid state emission [16,104]. Consequently, lateral triaryl-borane-based π-systems were realized with both high solid-state fluorescence efficiency and large Stokes shift for potential applications in OLEDs.

**Figure 13:**
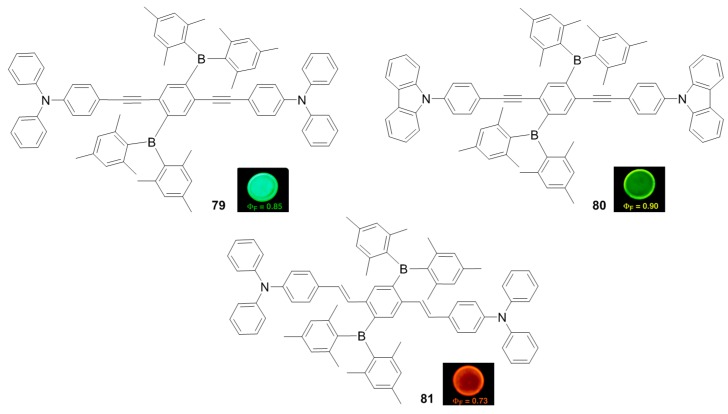
Triarylborane-substituted π-conjugated materials **79**–**81**.

In 2006, Yamaguchi and co-workers reported new D–A–D type quadrupolar molecules 79–81, containing two Mes_2_B pendants, oligo(phenyleneethynylene) (OPE) and oligo-(phenylenevinylene) (OPV) core units, and amino groups as electron-donors (Figure 13) [44]. These materials showed stability against air and water as well as thermal. Compound 79 exhibited large Stokes shift (Δν = 4491 cm^−1^ in cyclohexane) and strong positive fluorescence solvatochromism from 536 nm in cyclohexane to 601 nm in THF and 627 nm in methanol as a result of the intense ICT transition. Compounds 79–81 demonstrated green to reddish orange emission wavelengths of 562, 504 and 596 nm with *Φ_F_* of 0.90, 0.85 and 0.73 as solid state films, confirming that these structures were effective in suppressing the intermolecular interactions in solid state. As a result, they can be applied in optoelectronics as functional emissive materials with the intense solid state fluorescence properties.

**Figure 14:**
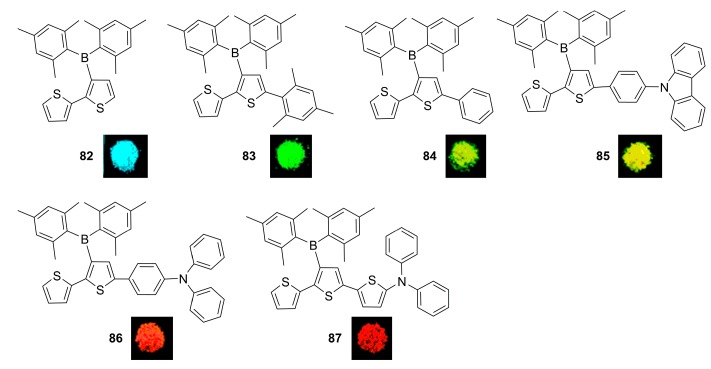
π-Conjugated materials **82**–**87** with Mes_2_B pendants.

Yamaguchi and co-workers investigated a series of 3-boryl-2,2′-bithiophene-based π-conjugated materials 82–87, containing different electron donating groups as new highly emissive systems (Figure 14) [16]. These materials achieved not only intense solid-state emissions, but also full colour emissions covering a wide range from blue (477 nm for 82) to deep red (660 nm for 87). In addition, all the compounds exhibited large Stokes shifts and efficient solid state emissions by ICT transitions from thiophene moiety to the boron pendant.

**Figure 15:**
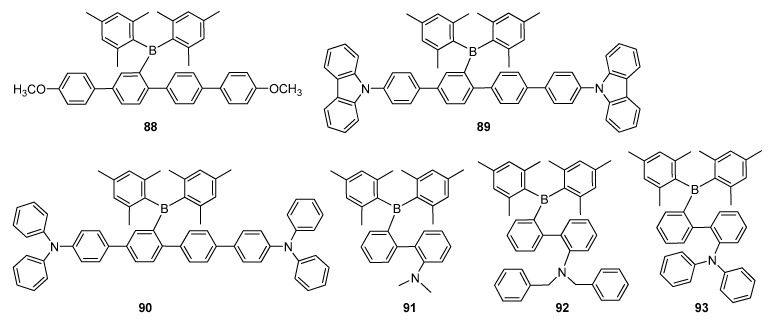
π-Conjugated materials **88**–**93** with Mes_2_B pendants.

To achieve an intense solid-state emission, Zhao and co-workers designed a new family of *p*-quaterphenyls 88–90, laterally substituted with bulky electron-deficient Mes_2_B groups (Figure 15) [105]. The *p*-quaterpheyl backbone displayed very limited conjugation, which was expected to hinder the red shift, so thus, they provided in blue light area with emission maxima wavelengths ranging from 446 to 473 nm. In addition, all the lateral boryl substituted *p*-quaterphenyl derivatives indicated an intense fluorescence in spin-coated films with excellent *Φ_F_* of 0.99 for 88, 0.83 for 89 and 0.99 for 90 due to their absence of the intermolecular interaction in solid state as well as a large Stokes shift induced by the ICT transition. In particular, compounds 89 and 90, containing carbazole and TPA groups on a π-conjugated framework, could be employed as promising ambipolar transporting blue emitters in OLED applications, owing to their high thermal stabilities, good oxidation−reduction properties and excellent solid state fluorescence efficiencies.

The convincing results obtained through the use of lateral boryl substituted π-systems, allowed Zhao’s group to report new CT-emitting triarylborane π-systems 91 and 92, in which boryl and amino groups were located at the lateral *o*,*o*′-positions of a biaryl framework (Figure 15) [104,106,107] Compound 91 expectedly displayed very intense fluorescence with green emission maximum at 523 nm in solid state (*Φ_F_* = 0.86 as a spin-coated film) and large Stokes shift of 215 nm. On the other hand, compound 92 was very emissive and significantly blue-shifted (Δλ_em_ = 62 nm as a spin-coated film), whereas its fluorescence efficiency was recorded as moderate (*Φ_F_* = 0.35 as a film) due to the pronounced steric bulk effect of NBn_2_ compared to methyl units.

The same group synthesized triarylborane-based biphenyl derivative 93, containing Mes_2_B and TPA at the lateral *o*,*o*′-positions of the biphenyl backbone (Figure 15) [108]. It showed a higher triplet energy (E_T_ = 2.57 eV) compared to its normal linear regioisomer, *p*,*p*′-NPh_2_ (E_T_ = 2.28 eV) as a result of the well-separated HOMO and LUMO levels and, thus, resulting in a small Δ*E*_ST_. Since the typical blue phosphorescent emitter FIrpic had E_T_ of 2.62 eV, molecule 93 was asserted to be provided as a host material in blue and green phosphorescent OLEDs. The phosphorescent OLED devices based on 93 achieved excellent performances with EQEs of 15.3 and 22.4%, *η_C,max_* of 34.5 and 84.2 cd·A^−1^ and power efficiencies of 31.4 and 76.6 lm·W^−1^ for blue and green phosphorescent OLEDs, respectively.

## 3. Triarylborane Based Polymers

D–A type conjugated polymers, having highly electron-deficient triarylborane groups were studied to produce new conjugated materials [15,28]. Such systems show intriguing properties such as low-lying LUMOs, easy reduction, and bathochromic shifts in of the absorption and emission spectra. However, bulky aryl groups, such as mesityl, have been generally used as the substituents on boron to improve the stability and maintain the electron-deficient character of boron.

**Figure 16:**
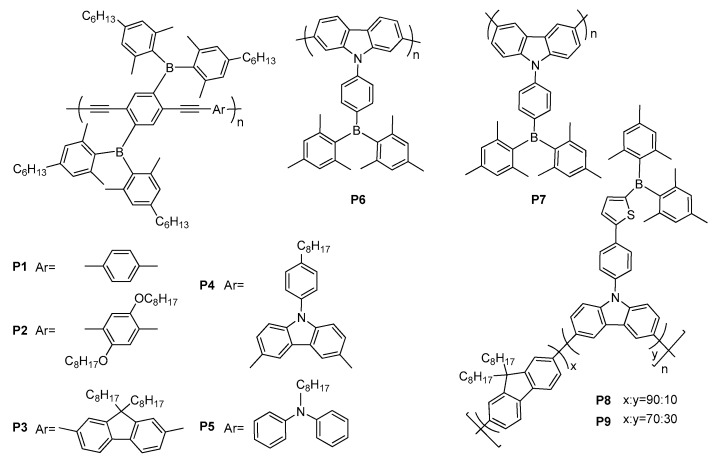
Triarylborane-substituted π-conjugated polymers **P1**–**P9**.

Triarylborane-based polymers were designed by considering two kind of D–A systems, namely, the boron group (i) as a pendant on the side chain and (ii) as a backbone unit in the main chain [28]. Although triarylborane-containing small molecules have been successfully developed as light emitters, only their few light emitting polymers have been suggested for PLED applications [7,109,110,111,112,113]. In 2007, Yamaguchi and co-workers reported a series of new emissive polymers **P1**–**P5** possessing two diarylboron pendants on the side chains with various co-monomers and poly-(arylene ethynylene)s as building units (Figure 16) [111]. All the polymers were characterized to be stable toward air and water and highly soluble in common organic solvents. **P1**–**P5** demonstrated a blue-greenish to yellow intense emissions in benzene with quantum yields ranging from 0.87 to 0.98 and in solid state (*Φ_F_* = 0.36–0.54), which allowed them to be promising functional building blocks for emissive π-conjugated materials. Reitzenstein et al. investigated the influence of connection positions of 2,7- and 3,6-linked polycarbazoles **P6** and **P7,** containing a triarylborane acceptor, on their absorption and emission properties (Figure 16) [113]. 3,6-Linkage in the main chain of **P7** had a strong effect on its optical properties resulting in better *Φ_F_* of 28% and 15% as solid powder and as a film, respectively, due to a low-lying fluorescent CT state compared to the optical properties of 2,7-linked polycarbazole **P6**. Polymer **P7** was reported to be a blue emitter with CIE coordinates of (0.17, 0.21).

Recently, Chen et al. reported carbazole/fluorene copolymers **P8** and **P9**, in which carbazoles were connected with Mes_2_B as pendants through phenyl-thiophene spacers to be used as a light-emitting layer in blue light-emitting diodes (Figure 16) [114]. They showed blue emission maxima at 456 and 462 nm with high *Φ_F_* of 72 and 69% emerging from their fluorescent ICT between the Mes_2_B and carbazole groups. The OLED devices fabricated using **P8** and **P9** exhibited blue emissions with CIE coordinates (0.16, 0.11) and (0.16, 0.13), respectively. Their V_on_ values of 6.5 and 5.5 V, maximum brightness of 445 and 414 cd·m^−2^ and highest luminescence efficiencies of 0.51 and 0.34 cd·A^−1^ were reported, respectively.

**Figure 17:**
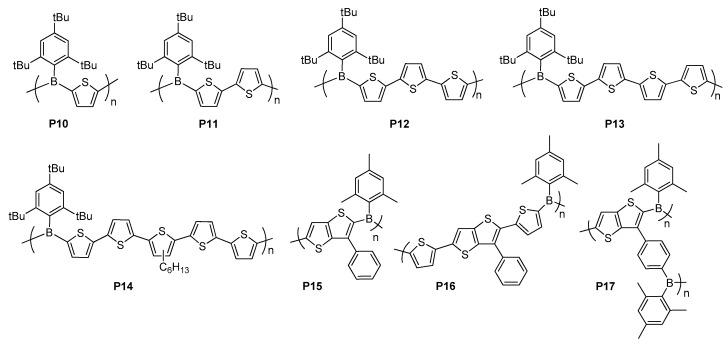
Triarylborane-based π-conjugated polymers **P10**–**P17**.

Jäkle and co-workers reported a series of polymers **P10**–**P14** having tributylboron units as an electron acceptor in the conjugated polythiophene main chain (Figure 17) [115]. All the polymers exhibited excellent long-term chemical stabilities against air and moisture and remarkable thermal and oxidative stabilities as well as good solubility in common organic solvents. With changing the length of the π-conjugated oligothiophene spacer between the boron units, the materials demonstrated tuned emission colours from blue to deep orange with fluorescence maxima ranging between 495 and 657 nm in solid state. As a result, triarylborane-based polymers were identified as promising materials for the improvement of optoelectronic applications owing to their controllable electronic structures and photophysical properties.

Ozturk and co-workers developed new emissive D–A type copolymers **P15**–**P17**, containing TT as an electron donor and MesB as a π-acceptor in the main chain for optoelectronic applications (Figure 17) [116,117]. These polymers displayed strong fluorescence with blue to yellow emissions between 441 and 566 nm in solid state. Particularly, the polymer light emitting diode (PLED) devices based on **P16** and **P17** exhibited pure white emission with EL maxima at 620 and 677 nm with CIE coordinates of 0.32, 0.35 and 0.34, 0.34, respectively, although their fabricated devices showed poor performances with V_on_ of 8.1–11 V, luminous efficiency of 0.035–0.01 and EQEs of varying from 0.007 to 0.025%. Nevertheless, with this study, use of boron as a strong electron acceptor and TT as an electron donor to achieve white light emitting polymers was demonstrated for the first time.

## 4. Conclusions

Triarylboranes have been utilized as strong acceptors with donors forming D–A type conjugated luminescent small molecules and polymers which have been used as emitting, hole transporting and electron transporting layers to improve the performances of OLEDs. They have been considered to provide low energy ICT transition bands upon photoexcitation and, thereby, enhanced solvatochromic fluorescence emission. Triarylboranes were also recognized to increase the chemical (against oxygen and moisture) and thermal stabilities by diminishing the LUMO energy levels and, consequently, rendering small HOMO–LUMO gaps, which are a prerequisite for achieving red/NIR emissive materials. Insertion of an arylboron between donor units or as a terminal group in the main chain as well as its use as a pendant were demonstrated to reinforce the quantum yields and, hence, augment OLED performances. However, the effect of lateral arylborons was detected to be much more pronounced with respect to those inserted into the main chain. The green phosphorescent OLED device based on **93**, possessing Mes_2_B pendant, for example, provided excellent performance with an EQE of 22.4%, whereas a red phosphorescent OLED device containing **66** with Mes_2_B terminal units gave 20.7% of EQE. Triarylboranes were also probed as an emitting layer in TADF processes, a newly evolving subject, leading to confirmation of their potential use in OLED applications. Applying the TADF process, up to 22.8% of EQE was reached with compound **70**, having a simple D–A system. All the performed investigations on triarylboranes were briefly overviewed in this report, which indicates that triarylboranes have high potential for its application in OLED technology.
